# Supporting and Retaining Advanced Trainees and New Consultants in AWP

**DOI:** 10.1192/bjo.2026.11316

**Published:** 2026-06-30

**Authors:** Harriet Greenstone, Sarah Price, Theresa Tattan, Angelika Luehrs

**Affiliations:** Avon and Wiltshire Mental Health Partnership Trust, Bristol, United Kingdom

## Abstract

**Aims:**

High quality patient care in mental health services relies on a robust psychiatric workforce. However, unfilled posts and high staff turnover can become entrenched problems. Here we describe a unique and innovative strategy developed in Avon and Wiltshire Mental Health Partnership Trust, developed to support and retain advanced trainees and new consultants in the trust. This aimed to support consultants in their first 3–4 years of appointment and to develop consultant roles within the trust that would appeal to trainees.

**Methods:**

The strategy was developed in response to recruitment and retention challenges. The strategy consisted of five elements, including 1) Roll-out of new consultant-specific induction, including a bespoke leadership development programme and leadership coaching for new consultants. 2) Funding and support of a research fellow to conduct qualitative research around experiences of the transition period between higher training and becoming a consultant. 3) Work around support and wellbeing of medical staff. 4) Development of a mentoring programme. 5) Development of compassionate leadership in the trust. Further details of each of the above interventions are described, including challenges faced and how these were overcome. Feedback and outcome measures are then described.

**Results:**

Arising from our work locally, we have generated several recommendations to support and retain advanced trainees and consultants within mental health trusts. We share experiences from developing and implementing these interventions locally. Sharing this information may help other mental health trusts nationally in addressing similar challenges.

**Conclusion:**

We describe an innovative local strategy, developed to support and retain advanced trainees and new consultants in the trust. Future areas for development could include a) sharing successful approaches more widely with other trustsand b) further consolidation of local activities. Further consolidation locally would include promoting more widespread uptake of mentoring, including this being offered to advanced trainees in the two years prior to their completion of training, and continuing to develop cross-trust mentoring with neighbouring trusts.

